# Structure of *Bacillus subtilis* Ku-mediated DNA synaptic complex

**DOI:** 10.1093/nar/gkaf1036

**Published:** 2025-10-21

**Authors:** Whan-Jong Kim, Jieun Kim, Mingyu Jo, Youngjin Kim, Min-Sung Kim

**Affiliations:** Department of Life Sciences, Pohang University of Science and Technology, Pohang, Gyeongbuk, 37673, Republic of Korea; Department of Life Sciences, Pohang University of Science and Technology, Pohang, Gyeongbuk, 37673, Republic of Korea; Department of Life Sciences, Pohang University of Science and Technology, Pohang, Gyeongbuk, 37673, Republic of Korea; Department of Life Sciences, Pohang University of Science and Technology, Pohang, Gyeongbuk, 37673, Republic of Korea; Department of Life Sciences, Pohang University of Science and Technology, Pohang, Gyeongbuk, 37673, Republic of Korea

## Abstract

DNA double-strand breaks (DSBs) pose a severe threat to genomic integrity, and cells rely on two major pathways for repair: homologous recombination and non-homologous end joining (NHEJ). While eukaryotic NHEJ requires a multi-component assembly including the Ku70/80 heterodimer, bacterial NHEJ operates with a simpler toolkit comprising a Ku homodimer and the multifunctional LigD. Despite this simplicity, the mechanism by which broken DNA ends are bridged together has remained unclear in bacterial NHEJ. Here, we present a cryo-electron microscopy structure of the *Bacillus subtilis* Ku (bsKu)–DNA complex at 2.74 Å resolution, capturing two blunt DNA ends bridged by a Ku protein alone. Supported by further biochemical assays, we propose an integrated model in which oligomeric arrays of Ku homodimers bridge and stabilize two DNA ends, facilitating efficient DSB repair in *Bacillus subtilis*. This work reveals a bsKu-mediated DNA bridging mechanism distinct from the eukaryotic system and provides critical structural insight into prokaryotic DNA repair.

## Graphical Abstract

**Graphical Abstract:**
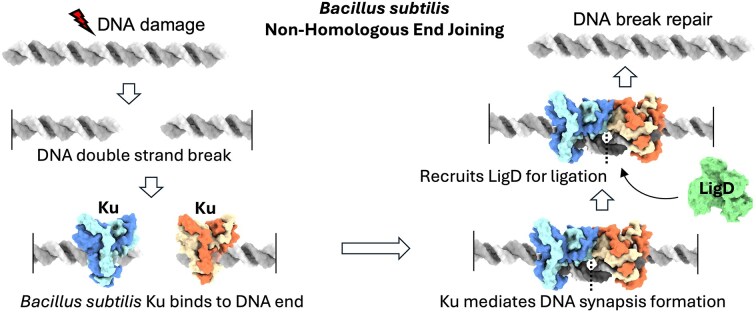

## Introduction

DNA double-strand breaks (DSBs) represent one of the most severe forms of DNA damage, posing a serious threat to cell viability [1–3]. To counter this, most organisms have evolved dedicated repair pathways, with non-homologous end joining (NHEJ) and homologous recombination (HR) serving as the primary mechanisms for DSB repair [4–7]. HR, an error-free mechanism for repairing DSBs, is initiated by the generation of long 3′ single-stranded DNA overhangs at DNA break sites, which act as probes to search for a homologous sequence on the sister chromatid with the assistance of HR-associated protein factors such as RPA and RAD51 [8]. In contrast, NHEJ operates by directly joining two DNA ends without the need for a homologous template, making it inherently error-prone [9]. For successful repair, the two broken DNA ends must be brought into close proximity to form a DNA end synapsis before ligation can occur. This synapsis-forming step is critical and is facilitated through a coordinated, multi-component, and stepwise process [10].

First identified in *Homo sapiens*, eukaryotic NHEJ is initiated by the loading of the heterodimeric Ku70/80 complex onto DNA break ends [11]. The Ku70/80 heterodimer is the first responder to recognize DNA break ends, where it protects the ends from further degradation [12]. Subsequently, Ku70/80 functions as a scaffold to recruit key NHEJ factors such as DNA-PKcs, PAXX, XLF, and the XRCC4–Ligase IV complex [13–15]. The formation of broken DNA end synapsis through a multi-component assembly is well illustrated by the three-dimensional (3D) structural analysis of the eukaryotic synaptic complexes [16]. After forming synapsis, these factors coordinate a multistep process culminating in DNA end ligation [17, 18].

The first bacterial NHEJ system was subsequently identified in *Bacillus subtilis*, consisting of a simpler set of core components than the eukaryotic system, primarily the Ku homodimer and LigD [19–22]. Similar to its eukaryotic counterpart, the bacterial Ku homodimer acts as the first responder to DSBs, recognizing the damaged ends and recruiting LigD to the break sites [1, 23]. LigD is a multifunctional protein comprising ligase, esterase, and polymerase domains. The order and organization of these domains vary across bacterial species. For example, *Bacillus subtilis* LigD contains only the ligase and polymerase domains [24].

Interestingly, the Ku proteins of bacteria and eukaryotes differ not only in molecular weight but also in domain composition. For instance, the bacterial Ku homodimer has a molecular weight of ∼80 kDa, while the eukaryotic Ku70/80 heterodimer is significantly larger, at around 150 kDa [25]. These structural differences suggest that bacterial and eukaryotic Ku proteins may perform distinct functions beyond their conserved role in recognizing broken DNA ends. A possible role for the polymerase domain of LigD in mediating DNA end synapsis has been proposed [26]. However, the molecular mechanism by which two DNA ends are brought together to form a synapsis during bacterial NHEJ remains poorly understood.

Recently, Baral *et al.* demonstrated that mycobacterial Ku alone can form oligomeric complexes in a DNA-dependent manner [27]. Meanwhile, another study showed that, unlike the eukaryotic Ku70/80 complex, the *Bacillus subtilis* Ku (abbreviated as bsKu) homodimer is capable of transiently bridging broken DNA ends, forming a synaptic complex that lasts ∼2 s *in vitro*, as demonstrated by single-molecule analysis. This bsKu-mediated synapsis is further stabilized upon the addition of LigD [28]. Together, these findings imply that bacterial Ku alone may actively contribute to the formation of DNA end synapsis during NHEJ.

To date, no 3D structural information is available for the bacterial Ku protein, either in its apo form or in complex with DNA, aside from an AlphaFold-predicted model. This lack of structural insight limits our understanding of how Ku facilitates the synapsis of broken DNA ends and cooperates with LigD to complete the ligation process in bacteria.

We present a cyro-electron microscopy (cryo-EM) structure of the bsKu–DNA complex at moderately high resolution (2.74 Å), revealing that bsKu alone mediates DNA synapsis by bridging two broken DNA ends, specifically in the context of blunt-end ligation. The structure also reveals two specialized DNA-binding motifs within bsKu.

Additionally, we conducted biochemical assays to further elucidate the mechanism by which bsKu recognizes and bridges DNA ends. Collectively, our results demonstrate that bsKu operates via a mechanism fundamentally distinct from its eukaryotic counterpart, employing a unique mode of DNA end bridging during NHEJ. Based on these findings, we present an integrated model illustrating how multiple Ku homodimers cooperate to mediate synapsis between two broken DNA ends in the process of bacterial NHEJ.

## Materials and methods

### Protein expression and purification

The DNA sequence of the *Bacillus subtilis* Ku core (UniProt ID: O34859, residues Met18–Asn244) was cloned into a modified pPROEX-HTb vector containing an N-terminal 8× His–MBP tag, which is cleavable by PreScission protease. The recombinant plasmid was transformed into *Escherichia coli* BL21 (DE3). Protein overexpression was induced with 0.5 mM isopropyl β-d-1-thiogalactopyranoside (IPTG) at 37°C for 5 h. Cells were harvested and resuspended in a buffer containing 20 mM HEPES (pH 7.5), 0.5 M KCl, and 1 mM TCEP, supplemented with 0.5 mM PMSF, cOmplete™ protease inhibitor cocktail (Roche), and 1 mg DNase I. Cells were lysed by sonication and centrifuged at 16 000 rpm for 1 h. The supernatant was filtered through a 0.45 μm filter and loaded onto a HisTrap column (Cytiva). The eluted protein was diluted fourfold with 20 mM HEPES (pH 7.5) and 1 mM TCEP, then applied to a HiTrap Q column (Cytiva) to remove contaminants. The N-terminal tag was cleaved by overnight incubation at 4°C with PreScission protease. The cleavage mixture was passed through a HisTrap column to remove the tag, and the flowthrough was further purified using a HiTrap Q column. The purified Ku protein was concentrated to 5–10 mg/ml, snap-frozen in liquid nitrogen, and stored at −80°C until use. All mutant proteins were purified using the same procedure as the wild type.

### DNA annealing

DNA strands were purchased from Integrated DNA Technologies (IDT) and annealed. The oligonucleotides were resuspended in 20 mM Tris (pH 8.0) and 50 mM NaCl, then desalted using a NAP DNA purification column (Cytiva). Complementary DNA strands were mixed at a 1:1 molar ratio. The mixture was denatured at 95°C for 10 min, then gradually cooled to 4°C at a rate of 1°C per min. The DNA sequences used in this study are listed in Supplementary Table S2.

### DNA binding test

The bsKu core protein was prepared in 20 mM HEPES (pH 7.5), 150 mM KCl, and 1 mM Dithiothreitol (DTT) following size-exclusion chromatography using a Superdex 75 10/300 GL column (Cytiva). The protein was mixed with double-stranded DNA (dsDNA) of various lengths at a 1:2 molar ratio (protein:DNA). The mixtures were incubated at 4°C for 1 h and then centrifuged. Samples were loaded onto a Superdex 75 10/300 GL column equilibrated with the same buffer (20 mM HEPES, pH 7.5, 150 mM KCl, 1 mM DTT). The presence of protein and DNA was confirmed by sodium dodecyl sulfate–polyacrylamide gel electrophoresis and denaturing Tris-Borate-EDTA (TBE)–urea gel electrophoresis, respectively.

### Electrophoretic mobility shift assay

bsKu core wild-type and mutant proteins were prepared in 20 mM HEPES (pH 7.5), 150 mM KCl, and 1 mM DTT. Double-stranded DNA (13 bp) was prepared at 500 nM by dilution in the same buffer. Reaction mixtures (20 μl total volume) contained 50 nM DNA substrate and varying concentrations of bsKu core protein. After incubation for 30 min at 4°C, 4 μl of loading buffer (60% glycerol with a trace amount of bromophenol blue) was added. Samples were loaded onto a 15% TBE native polyacrylamide gel and electrophoresed at 4°C at 200 V and 200 mA for 40–50 min in 0.5× TBE buffer. Gels were stained with SYBR Gold (Invitrogen) at room temperature for 15 min with continuous shaking. Images were obtained under 302 nm UV illumination.

### Cryo-EM sample preparation and data collection

The bsKu core protein was mixed with 13-bp dsDNA at a 1:2 molar ratio and incubated for 1 h at 4°C. The sample was injected onto a Superdex 75 10/300 GL column (Cytiva) equilibrated with 20 mM HEPES (pH 7.5), 150 mM KCl, and 1 mM DTT. Fractions containing the oligomeric Ku–DNA complex were collected and concentrated to 2 mg/ml. Before grid preparation, the concentrated sample was centrifuged to remove any insoluble material. A 3 μl aliquot of the sample was applied to a holey carbon grid (Quantifoil Cu R1.2/1.3, 300 mesh) that had been glow-discharged for 60 s at 15 mA using a PELCO easiGlow (Ted Pella Inc.). Grids were blotted using a Vitrobot Mark IV (Thermo Fisher Scientific) at a blot force of 2 for 3 to 7 s at 4°C and 100% humidity, then plunge-frozen in liquid ethane. Cryo-EM data were collected using a Titan Krios (Thermo Fisher Scientific) equipped with a BioQuantum K3 detector (Gatan). A total of 10 992 movies were collected at a total dose of 60 e^−^/Å^2^ over 60 frames.

### Data processing and model building

Image processing and map generation were performed using CryoSPARC v4.7.0 [29]. The overall workflow is summarized in Supplementary Fig. S2. Motion-corrected and CTF (contrast transfer function)-estimated micrographs were used for particle picking. An initial small set of particles was identified using both blob- and template-picking methods. These particles were used to train a Topaz model for automated particle picking. Junk particles were removed through several rounds of 2D classification, and the remaining particles were used to generate initial 3D reconstructions. Following heterogeneous refinement, only one class yielded a map consistent with a bsKu homodimer bound to DNA. This map was further refined using nonuniform refinement. The particles from this refinement were then used for a second round of Topaz-based particle picking to expand the dataset resulting in approximately three times more particles. These new particles were subjected to another round of 3D reconstruction and classification. Again, only one 3D class produced a clear map, which was further refined using nonuniform refinement. Due to the tendency of bsKu–DNA complexes to align in filamentous architecture arrangements, the resulting reconstruction showed extra densities from neighboring particles. To address this, custom masks were generated in ChimeraX [30] and used for particle subtraction and local refinement. The final reconstruction had a global resolution of 2.74 Å, as estimated by Fourier shell correlation (FSC) at the 0.143 threshold. For model building, an AlphaFold3 [31] predicted structure of the bsKu homodimer was used as a starting template. The model was manually fitted into the density in ChimeraX and adjusted in Coot [32]. Two bsKu homodimers were modeled and designated as chains A, B, C, and D. All residues were built except for residues 103–105 of chain B, due to weak density in the cryo-EM map. The bound double-stranded DNA was built manually in Coot. Final refinements were performed using real-space refinement in Phenix [33] and further fine-tuned in Coot.

## Result

### *Bacillus subtilis* Ku homodimers form linear oligomeric assemblies in a DNA-dependent manner

**Figure 1.**
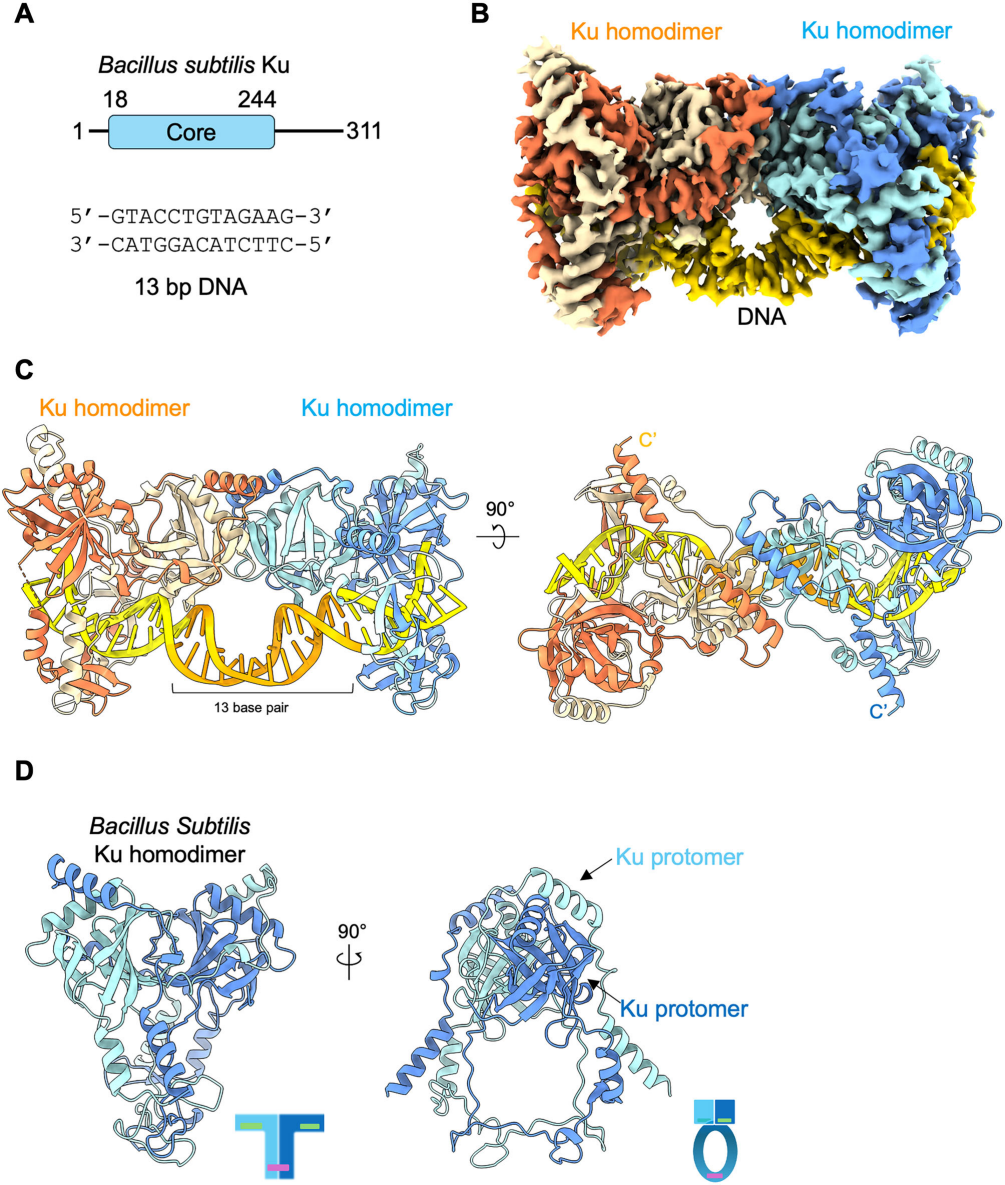
Cryo-EM structure of the *Bacillus subtilis* Ku–DNA complex. (**A**) Diagram of protein components of Ku and the sequence of DNA substrate. Numbers indicate amino acid positions. (**B**) Cryo-EM map of the bsKu–DNA complex. (**C**) Structural model of the bsKu–DNA synaptic complex. Two bsKu homodimers are bound to linear DNA molecules, with each protomer and DNA strand depicted in a distinct color. Among the modeled DNA, the segment corresponding to the added 13-bp substrate is shown in orange. (**D**) The structure of bsKu homodimer without DNA is shown, with each protomer colored pale blue and blue, respectively. A schematic representation of bsKu’s T-shaped architecture, featuring the central ring as the stem, is illustrated below.

Bacterial Ku contains a central DNA-binding core domain and a flexible C-terminal region, which has been shown to be involved in LigD interaction and DNA end anchoring [34]. Given that the C-terminal region is predicted to be intrinsically disordered, we generated an expression construct encoding only the core domain of bsKu (residues 18–244). This core bsKu protein was used for subsequent cryo-EM analysis and biochemical characterization (Fig. 1A).

To investigate the mechanism of bsKu binding to DNA, we assembled bsKu–DNA complexes using double-stranded DNA substrates of varying lengths and analyzed the resulting assemblies by size-exclusion chromatography. Among the tested substrates, the 13-bp DNA was the shortest length that promoted bsKu multimerization, as evidenced by a distinct elution profile (Supplementary Fig. S1). This multimeric bsKu-13 bp DNA complex was subsequently used for cryo-EM analysis (Supplementary Fig. S2). The resulting multimeric complexes were visualized by cryo-EM micrographs, revealing long filamentous assemblies ∼6 nm in diameter, displaying linear and curved morphologies (Supplementary Fig. S2A and B).

Three-dimensional reconstruction was performed using single-particle cryo-EM analysis, with a box size of 288 pixels selected to encompass the minimal repeating unit of the filamentous bsKu homodimer–DNA complex. The resulting cryo-EM map achieved a Gold-Standard Fourier Shell Correlation (GSFSC) resolution of 2.74 Å.

Details of data collection, model validation, and refinement statistics are provided in Supplementary Table S1. The cryo-EM data processing workflow, map validation metrics, and resolution estimation are shown in Supplementary Fig. S2.

Within the cryo-EM map, we were able to confidently build two bsKu homodimers comprising most residues from 18 to 244, along with 31 bp of DNA (Fig. 1B and C). The DNA density was well-resolved, with clearly distinguishable major and minor grooves. Model building was initiated using an AlphaFold3 [31]-predicted structure, and regions that did not fit the density were manually adjusted and refined.

The cryo-EM maps revealed two bsKu homodimers make contact with each other and are bridged by a single continuous DNA strand that threads through the central ring forming the stem of each baKu homodimer (Fig. 1B and C). Each bsKu homodimer adopts a T-shaped architecture with a ring forming the stem (Fig. 1D). Within each homodimer, the two bsKu protomers engage in extensive inter-subunit interactions, consistent with structural predictions from AlphaFold3 (Supplementary Fig. S3). In addition, each homodimer interacts with its neighboring homodimer via one arm of its T-shaped architecture, and this inter-dimer interaction is further stabilized by the continuous DNA strand bridging two baKu homodimers (Fig. 1C).

To our surprise, despite using a relatively short 13-bp DNA substrate, the cryo-EM map revealed a continuous and extended DNA density (Fig. 1B and C), with no clearly discernible break between individual DNA molecules, likely due to lower resolution in the DNA region of the map. Therefore, we modeled the DNA as an unbroken 31-bp double-stranded structure. The length corresponding to the 13-bp DNA segment used for cryo-EM sample preparation is indicated in Fig. 1C. This unexpected observation will be discussed in detail in a later section.

Additional density was observed at both ends of the modeled structure, which we interpret as originating from adjacent bsKu–DNA molecules within the filamentous assembly (Supplementary Fig. S2F). To improve particle alignment and enhance map resolution, we applied focused masking around the modeled structure during refinement. We consider this bsKu dimer-of-homodimers bound to DNA to represent the fundamental repeating unit in the filamentous assembly.

### Molecular basis of DNA recognition by bsKu

**Figure 2.**
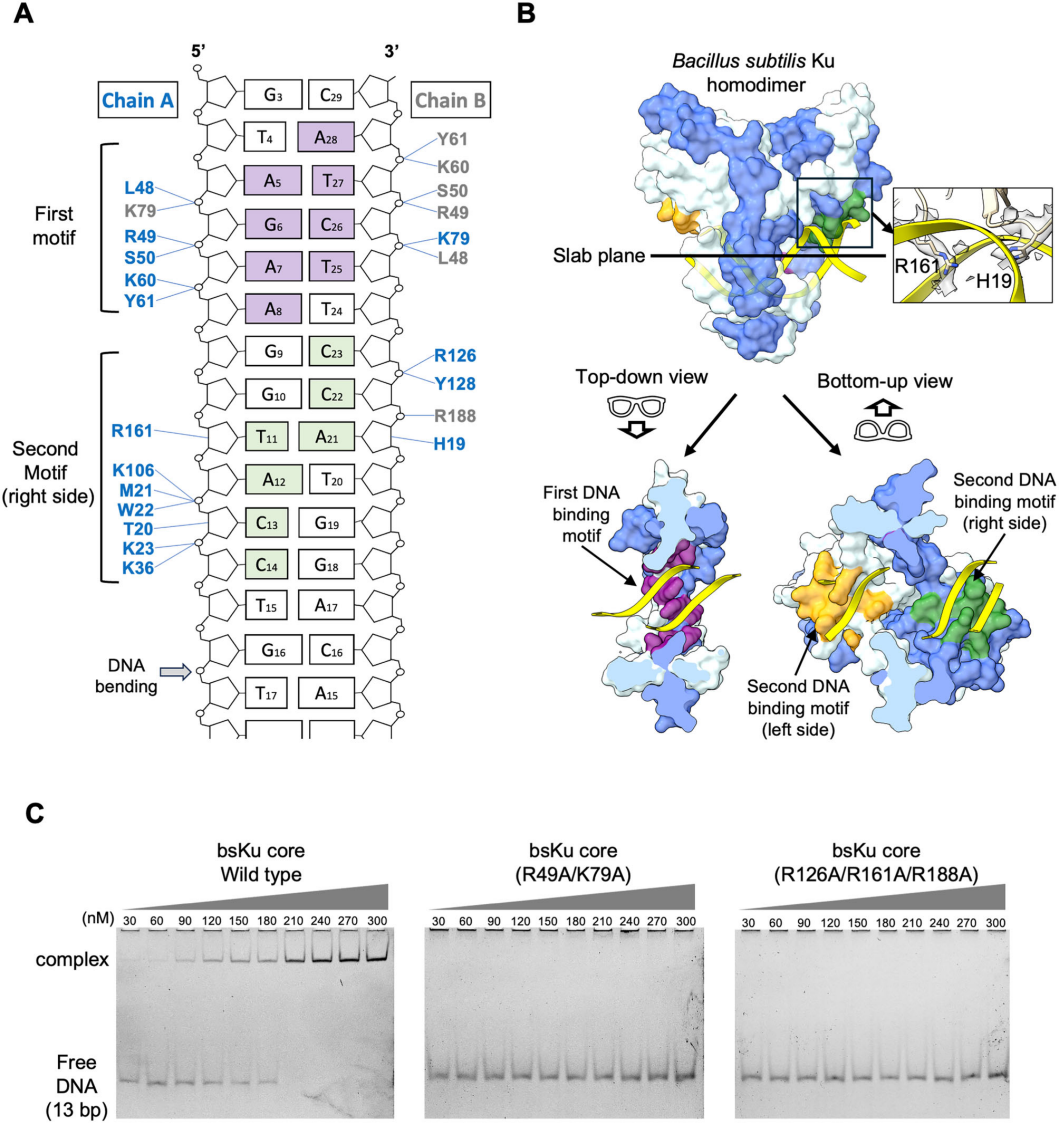
Two distinct DNA-binding motifs in the bsKu homodimer. (**A**) Details of the bsKu–DNA interactions at the first and second DNA-binding motifs are shown. The first motif interacts with 3–4 nucleotides from each DNA strand, while the second motif engages a continuous 6-bp region of DNA. The DNA nucleotides involved in binding are colored purple for the first motif and green for the second motif. The blue-colored residues are from chain A of Ku homodimer, corresponding to the blue protomer in panel (B), while the gray residues are from chain B, corresponding to the pale blue protomer in panel (B). (**B**) For clarity, only a single bsKu homodimer–DNA complex is shown in surface representation. The two protomers of bsKu homodimer are colored blue and pale blue, respectively, and DNA is shown in yellow. Top-down view: The first DNA-binding motif is located on the inner bottom side of the ring, which constitutes the stem of the T-shaped architecture, and is colored violet. Bottom-up view: In the T-shaped bsKu homodimer, the second DNA-binding sites are positioned along each arm, with the symmetric sites shown in orange and green, respectively. Key residues involved in minor groove recognition, H18 and R161, are highlighted along with cryo-EM density in the inset box. (**C**) EMSA analysis of bsKu core protein binding to 13-bp DNA, comparing the wild type with first DNA-binding motif mutant (double mutant: R49A/K79A) and second DNA-binding motif mutant (triple mutant: R126A/R161A/R188A). Protein concentrations used are indicated above each lane.

The relatively high resolution of our bsKu–DNA complex cryo-EM map (2.74 Å) enabled a detailed examination of the residues involved in DNA interaction. The bsKu homodimer engages DNA primarily through two distinct motifs for DNA binding. The first motif is positioned at the bottom of the inner ring and includes the residues L48, R49, S50, K60, Y61, and K79 from both protomers of the bsKu homodimer (Fig. 2A and B and Supplementary Fig. S4A). Notably, the positively charged side chains of R49 and K79 make direct contact with the DNA phosphate backbone, whereas the peptide backbone of other residues interacts with the DNA. Given that the ring region is formed by two bsKu protomers, the DNA interaction is symmetric, involving residues from both subunits. All residues within the first motif interact with the phosphate backbone of the DNA, indicating a nonsequence-specific mode of binding that is consistent with their moderate conservation across bacterial species (Supplementary Fig. S5). Interestingly, the upper part of the ring region does not participate in DNA interaction (Fig. 2B).

The second DNA-binding motif comprises residues H19, T20, M21, W22, K23, K36, K106, R126, Y128, and R161 from one protomer, along with R188 from the other protomer. Among these, H19 and R161 are deeply embedded within the minor groove of the DNA, suggesting a critical role in minor groove-specific recognition (Fig. 2A and B and Supplementary Fig. S4B). This second motif shows strong conservation of positively charged amino acid residues. All residues of the second motif, except M21 and W22, interact with DNA through their side chains. In contrast, the peptide backbones of M21 and W22 make contact with DNA, while their side chains contribute to stabilizing the protein structure (Supplementary Fig. S4B).

The second DNA-binding motifs are symmetrically positioned on both arm sides of the T-shaped architecture of Ku homodimer. Consequently, the Ku homodimer contains a single copy of the first DNA-binding motif and two copies of the second motif (Fig. 2B).

To validate this structural observation, we generated a double mutant in the first motif (R49A/K79A) and a triple mutant in the second motif (R126A/R161A/R188A), and examined their DNA binding by EMSA. Both mutants exhibited markedly reduced DNA binding (Fig. 2C), supporting our structural analysis.

Importantly, both DNA-binding motifs engage exclusively with the minor groove of the DNA, without contacting the major groove or bases. This selective mode of interaction is likely to facilitate the 1D sliding of Ku along the DNA duplex [34], enabling efficient DNA end search.

### The bsKu protein interface mediating filamentous oligomerization

**Figure 3.**
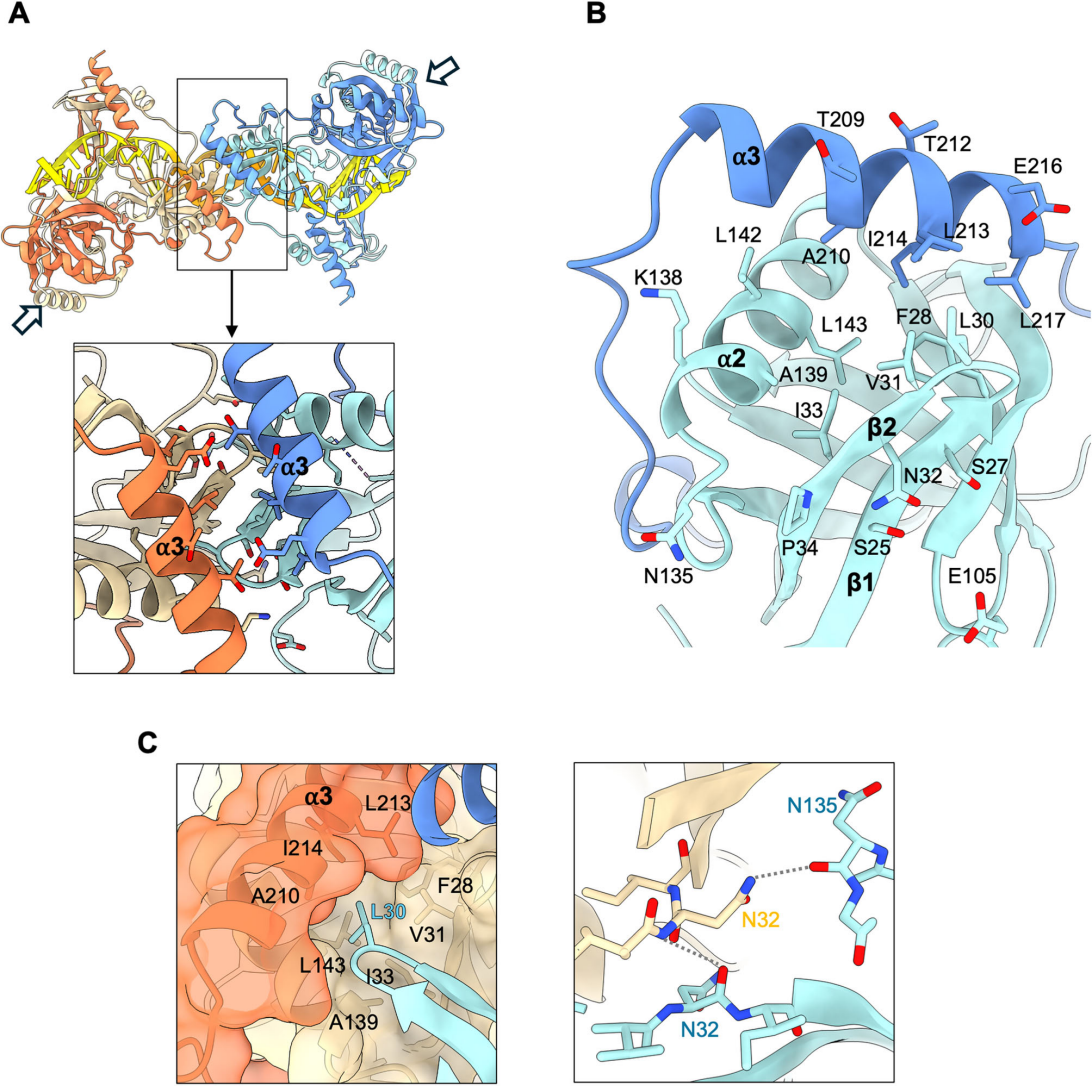
The binding interface is responsible for inter-bsKu homodimer interactions and oligomerization. (**A**) The contact interface between two bsKu homodimers is highlighted in the boxed region. Arrows indicate the binding site for the subsequent bsKu homodimer. (**B**) Because the binding interface is symmetric, only one binding site is shown for clarity. The interface residues are shown in a ball-and-stick representation. Most residues originate from one protomer (pale blue), while those from the α3 helix belong to the other protomer (blue). (**C**) Left: Hydrophobic interaction between L30 of one bsKu homodimer and a hydrophobic cleft on the neighboring bsKu homodimer. Right: N32 (sand color) forms hydrogen bonds with N32 and N135 of the neighboring bsKu homodimer (pale blue color).

We observed filamentous architectures formed by bsKu and DNA (Supplementary Fig. S2A and B), suggesting that the dimer of homodimers seen in our cryo-EM structure represents the fundamental repeating unit of bsKu polymerization. At the dimer–dimer interface, we identified several key residues that mediate intermolecular interactions (Fig. 3). Specifically, residues G24-S25, S27, and G29-P34 (located across the β1 and β2 strands), E105 (located in the loop between the α1 helix and β5 strand), N135-A139, and L142-L143 (within the α2 helix) from one protomer, as well as E206, T209-A210, T212-I214, and E216-L217 (within the α3 helix) from the other protomer, contribute to forming symmetric interfaces at both arms of the T-shaped architecture of bsKu. These interactions include hydrogen bonds, electrostatic interactions, and hydrophobic contacts. The total buried surface area at the interface is ∼957 Å^2^, as calculated using ePISA server [35].

Among these, L30, located in the β1–β2 loop, is deeply positioned within the cleft formed by hydrophobic residues of the neighboring bsKu homodimer, creating an anchor-like hydrophobic interaction (Fig. 3C, left). In addition, the side chain of N32 forms a hydrogen bond with the backbone carbonyl oxygen of N135, while the carbonyl oxygen of N32 forms another hydrogen bond with the peptide backbone nitrogen of the neighboring N32, thereby stabilizing the protein–protein interaction between bsKu homodimers (Fig. 3C, right). To validate these interactions, we generated L30G and N32E mutant bsKu proteins. Both mutants failed to form oligomeric complexes without significant loss of binding to 13-bp DNA fragment, demonstrating that these residues are essential for filamentous oligomerization (Supplementary Fig. S6A and B).

The residues forming this interface are well conserved across bacterial species (Supplementary Fig. S5), suggesting that Ku-mediated filamentous oligomerization may be a universal feature among bacteria. In particular, residues 29–34 (GLVNIP), located at the center of the interface, are highly conserved and have also been shown to be important for oligomerization in *Mycobacterium tuberculosis* Ku (residues 12–17, GLVNVP) [36]. Meanwhile, the residues within the α2 and α3 helices show low sequence conservation. Given that these less-conserved residues are positioned in the peripheral region of the interface, and considering the curved rather than straight arrangement of the filamentous complex observed in the EM images (Supplementary Fig. S2A and B), we infer that the inter-homodimer interface is not rigid but instead exhibits a degree of flexibility, allowing hinge-like motion at the dimer–dimer interface.

The bsKu interface-forming residues are not conserved in human Ku70 and Ku80, suggesting that filamentous oligomerization is a bacterial Ku-specific feature (Supplementary Fig. S7). Notably, in the absence of DNA, no bsKu multimerization was detected by size exclusion chromatography under physiological salt conditions. Multimerization was induced only upon binding to DNA of specific lengths (Supplementary Fig. S6A).

### Molecular mechanisms of synaptic complex formation at DNA end by bsKu

**Figure 4.**
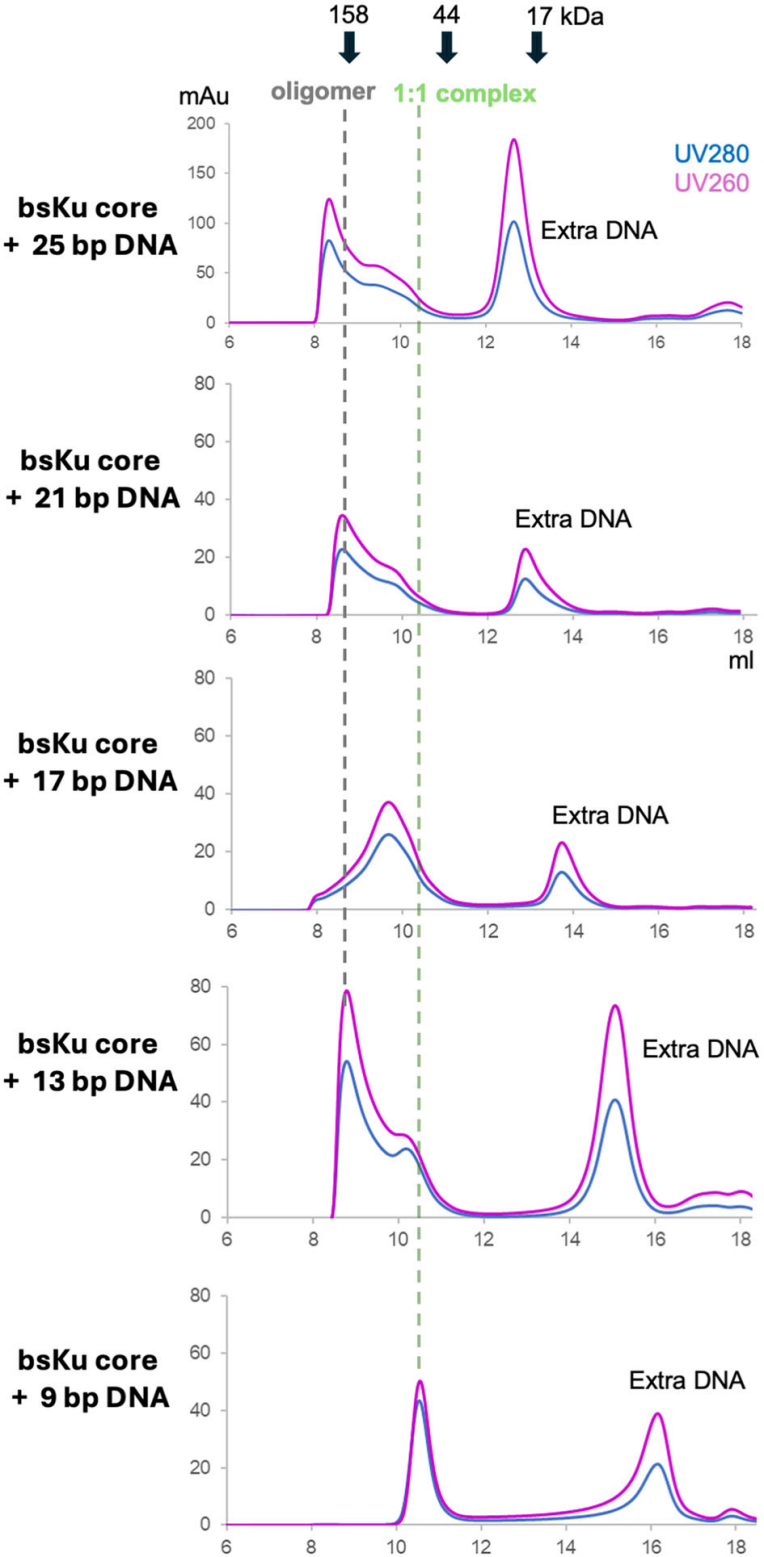
Oligomerization of bsKu is mediated by DNA of specific lengths. The bsKu core protein was incubated with DNA fragments of varying lengths, ranging from 9 to 25 base pairs, as labeled on the left. The sizes of the resulting bsKu–DNA complexes were analyzed by size-exclusion chromatography. Elution positions of molecular weight standards are marked with arrows at the top of the chromatograph (158 kDa = γ-globulin, 44 kDa = ovalbumin, and 17 kDa = myoglobin). The violet and blue lines represent UV absorbance at 260 nm and 280 nm, respectively. A higher absorbance at 260 nm compared to 280 nm indicates the presence of DNA. The dashed green line marks the position corresponding to a 1:1 stoichiometry of the bsKu–DNA complex. Complexes with longer DNA eluted slightly earlier due to their larger size. The dashed gray line indicates the position of oligomeric bsKu–DNA complexes.

As described earlier, to determine the optimal DNA length for the structural analysis of the bsKu–DNA complex, we screened double-stranded DNA substrates ranging from 9 to 25 base pairs (Fig. 4). The bsKu homodimer was able to bind DNA as short as 9 bp, as shown by size-exclusion chromatography. Interestingly, 13-bp DNA uniquely promoted the formation of higher-order bsKu assemblies, similar to the 21-bp DNA. In contrast, DNA fragments of intermediate length (17 bp) or shorter (9 bp) primarily formed a 1:1 complex with bsKu.

**Figure 5.**
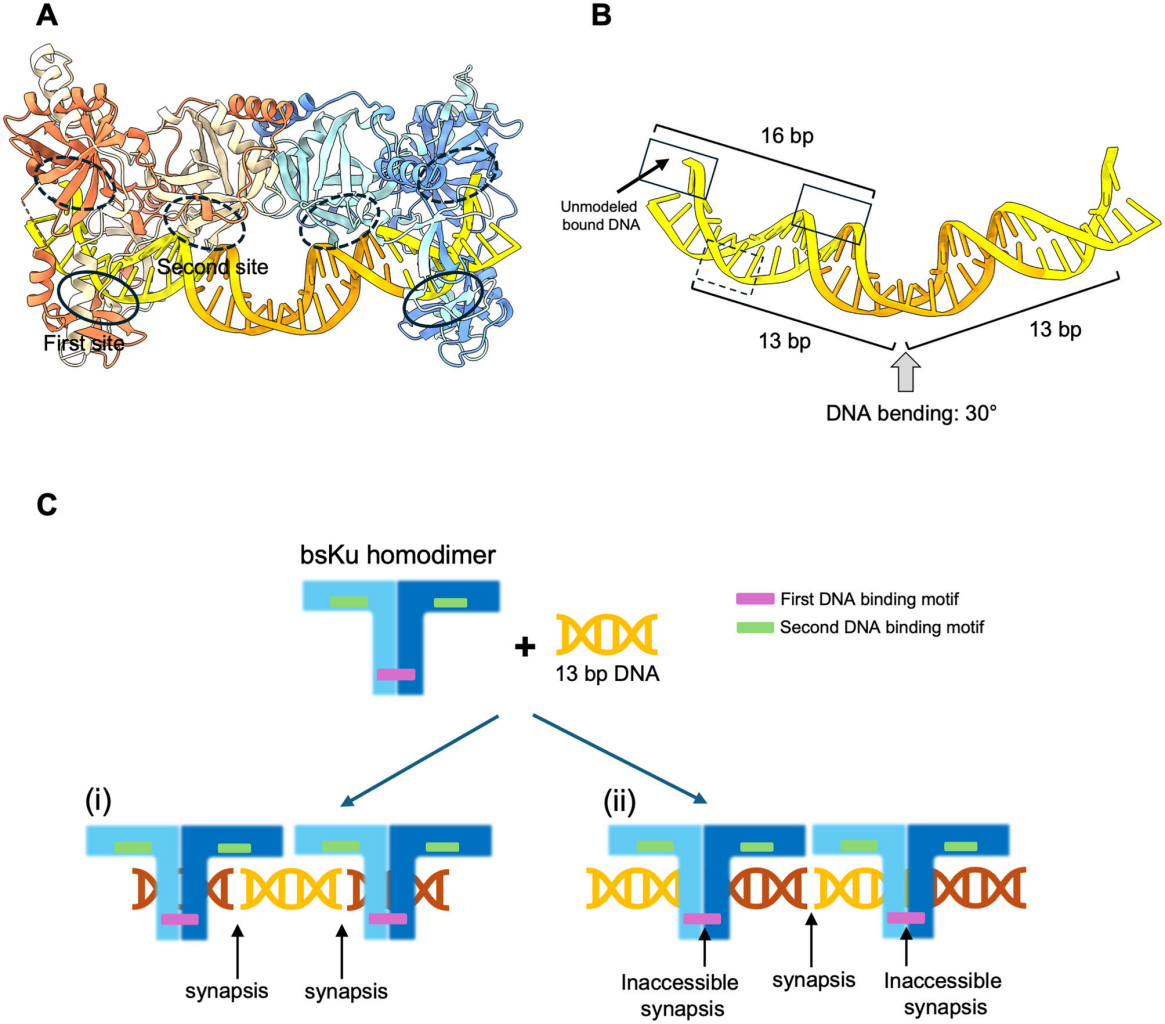
The *Bacillus subtilis* Ku homodimer interacts with DNA via two distinct DNA-binding motifs. (**A**) The first and second DNA-binding sites are indicated by solid and dashed circles, respectively. Each bsKu homodimer contains one first DNA-binding site and two symmetric second DNA-binding sites. (**B**) Only the bound DNA is shown, with the view orientation matching that of panel (A). Upon bsKu binding, the DNA is bent by ∼30°. The distance from the DNA bending point to the first DNA-binding motif (indicated by a dashed box) corresponds to 13 bp of DNA. The total length of DNA spanning a single bsKu homodimer is ∼16 bp. The solid-lined boxes highlight the positions of the second DNA-binding motifs located on the left and right arms of the bsKu homodimer. (**C**) Proposed model of synapsis formation by bsKu upon binding to 13-bp DNA. The first DNA-binding motifs are shown in violet, and the second DNA-binding motifs are colored green. Based on the DNA length observed in our cryo-EM structure, two potential modes of 13-bp DNA binding are suggested. In the first mode, DNA synapsis occurs within the second DNA-binding motif (labeled i, left). In the second mode, DNA synapsis forms at the DNA bending site between two bsKu homodimers (labeled ii, right). The 13-bp DNA fragments are colored differently to enhance visual clarity.

Based on our cryo-EM structure, a single bsKu homodimer engages ∼16 bp of DNA (Fig. 5A and B). Therefore, when DNA fragments longer than 21 bp are added, the DNA can extend beyond the two DNA-binding motifs of a bsKu homodimer and interact with an additional Ku molecule, thereby promoting oligomerization regardless of DNA end synapsis formation. Meanwhile, DNA fragments of 17 bp are sufficient to occupy both the first and second DNA-binding motifs within a single bsKu homodimer but are not long enough to accommodate an additional Ku homodimer, which in turn inhibits multimerization. DNA fragments shorter than 9 bp are too short to bridge between two Ku homodimers. In either case, the configuration is unfavorable for Ku-mediated DNA synapsis formation.

Interestingly, for the 13-bp DNA, two possible binding models can be proposed based on the DNA structure observed in our cryo-EM structure, as illustrated in Fig. 5C. In the first model, the 13 bp is long enough to interact with half of second DNA-binding motifs on each homodimer, resulting in its acting as a bridge between two bsKu homodimers. In this case, DNA synapsis happens in the middle of second DNA binding site of bsKu. In the second model, the 13-bp DNA engages with half of the first DNA-binding motif and fully occupies the second DNA-binding motif on one arm of Ku homodimer. This arrangement results in the formation of a single DNA synapsis between two Ku homodimers, where DNA bending is observed in our structure (Fig. 5B and C).

Since bacterial Ku does not bind single-stranded DNA [37], we also examined whether DNA substrates containing single-strand overhangs could promote multimeric bsKu complex formation. To test this, we used an 11-bp DNA substrate with a 2-nucleotide overhang at one end. Although bsKu still bound efficiently to this DNA substrate, it did not induce oligomerization (Supplementary Fig. S6A). This suggests that Ku-dependent DNA synapsis specifically requires blunt ends at both DNA termini being joined.

When using longer DNA substrates (over 21 bp), we can detect the formation of filamentous complexes. However, it is difficult to determine whether bsKu is simply accumulating along the DNA strands or actually facilitating synapsis between DNA ends. In contrast, our use of a short 13-bp DNA substrate, which is shorter than the full DNA binding surface of a single bsKu homodimer, provides a more direct indication of DNA synapsis formation by bsKu.

## Discussion

To date, the DNA repair and mutagenesis pathways in the Gram-negative bacterium *Escherichia coli* are among the most extensively characterized. In contrast, among Gram-positive bacteria, *Bacillus subtilis* is widely used as a model organism to study a variety of bacterial cellular processes. *Bacillus subtilis* can endure a broad range of environmental stress conditions by forming a resilient haploid endospore, which can later germinate and reinitiate vegetative growth [38]. Notably, during the haploid phase of *Bacillus subtilis*, NHEJ serves as the primary DNA-double strands break repair mechanism supporting spore germination [23].

Our structural and functional analyses offer a new perspective on bsKu-mediated broken DNA-end synapsis formation during bacterial NHEJ. The cryo-EM structure of bsKu–DNA complex presented here reveals, for the first time, high-resolution molecular details of Ku-dependent synapsis formation. Based on these observations, we propose a model for the bsKu-dependent synaptic complex of blunt DNA ends. We further suggest that linear oligomerization of Ku following DNA end binding may restrict the flexibility of DNA ends, thereby facilitating their proper alignment and pairing for efficient DNA synapsis formation.

While preparing this manuscript, we became aware of a preprint on bioRxiv by Joydeep Baral *et al.* [36], which also reported the formation of linear-shaped oligomers of *Mycobacterium tuberculosis* Ku proteins upon DNA binding. Their findings align with and further support our observations, suggesting that a conserved mechanism of Ku-mediated DNA synapsis may operate across bacterial species during the NHEJ process.

We also observed DNA length-specific oligomerization of bsKu–DNA complex, suggesting the existence of an optimal DNA length that promotes Ku-dependent synapsis formation *in vitro*. In particular, short DNA fragments of 13 bp were just ideal to induce bsKu oligomerization, indicating true synapsis formation rather than nonspecific accumulation of bsKu along longer DNA strands.

In our cryo-EM study, we used a C-terminus truncated form of bsKu. However, previous studies have suggested that the C-terminal region plays a functional role, potentially through interactions with LigD [34]. This extended region is located at the stem of the T-shaped Ku homodimer, near the DNA end synapsis site, positioning it well to facilitate the recruitment of LigD to the DNA synapsis. Nonetheless, a detailed understanding of the interactions between Ku and LigD will be essential for fully elucidating the bacterial NHEJ mechanism.

Compared to mammalian Ku, bsKu contains only the minimal core DNA-binding domain along with a unique C-terminal extension that interacts with LigD (Supplementary Fig. S8A and B). Human Ku70 and Ku80 possess an additional N-terminal von Willebrand factor A (vWA) domain, which acts as a secondary determinant for heterodimerization and mediates interactions with other proteins [39, 40]. The C-terminus of Ku70 is flexible and includes a SAP domain, which helps anchor the Ku heterodimer at DNA ends, while the C-terminal region of Ku80 is responsible for interacting with DNA-PKcs [41].

Since NHEJ involves multiple steps leading to final DNA end ligation [42], such as DNA end processing and polymerization, it is possible that our cryo-EM structure represents a specific intermediate or one of several alternative pathways within the bacterial NHEJ process. For example, previous studies have suggested that DNA synapsis can also be mediated by the polymerase domain of LigD [26]. However, that structure describes synapsis formation between DNA ends with 3′ overhangs and does not explain how synapsis is achieved with blunt-ended DNA. Therefore, a coordinated mechanism that adapts to the specific nature of the DNA termini is likely required.

In this study, we identified a distinct capacity of bsKu to mediate the synapsis of broken DNA ends. The residues involved in bsKu oligomerization are well conserved, suggesting that this oligomerization mechanism may be universal among bacterial species. Based on our structural observations, we propose two possible models for bsKu-mediated DNA end synaptic complexes (Fig. 5C). Further experimental investigation will be necessary to determine which configuration is more favorable for subsequent LigD-mediated ligation. It also remains to be clarified whether single-stranded DNA overhangs can be directly ligated in a Ku-dependent manner or whether DNA end-processing activities of LigD, such as polymerase and/or esterase functions, are required first to convert the DNA ends to blunt termini prior to ligation. Additionally, our findings demonstrate that the bacterial Ku-mediated NHEJ mechanism is mechanistically distinct from its eukaryotic counterpart, highlighting its potential as a novel target for antimicrobial drug development, especially through disruption of its unique DNA binding mode or bacterial Ku oligomerization interface.

## Supplementary Material

gkaf1036_Supplemental_File

## Data Availability

For bsKu-13 bp DNA synaptic complex, the cryo-EM density map has been deposited in the Electron Microscopy Data Bank (EMDB) under the ID EMD-65215. The coordinate has been deposited in the Protein Data Bank (PDB) under the ID PDB-9VNQ. This paper is linked to: https://doi.org/10.1093/nar/gkf1418.
