# Supplementary material for: Structure of Bacillus subtilis Ku-mediated DNA synaptic complex
Source: Nucleic Acids Res. 2025 Oct 21;54(1):gkaf1036. doi: 10.1093/nar/gkaf1036 (PMC12784961; doi:10.1093/nar/gkaf1036)
Supplement: gkaf1036_Supplemental_File [file gkaf1036_supplemental_file.pdf]

Supplementary Information for

Structure of *Bacillus subtilis* Ku mediated DNA synaptic complex

Whan-Jong Kim[1], Jieun Kim[1], Mingyu Jo[1], Youngjin Kim[1] and Min-Sung Kim[1*]

[1]Department of Life Sciences, Pohang University of Science and Technology, Pohang, Gyeongbuk, Republic of Korea
*Corresponding author: Min-Sung Kim (Email: cggs@postech.ac.kr)

This supplementary file inclues
Supplementary Figure 1-8
Supplementary Table 1-2
Supplementary References

Supplementary Figure 1

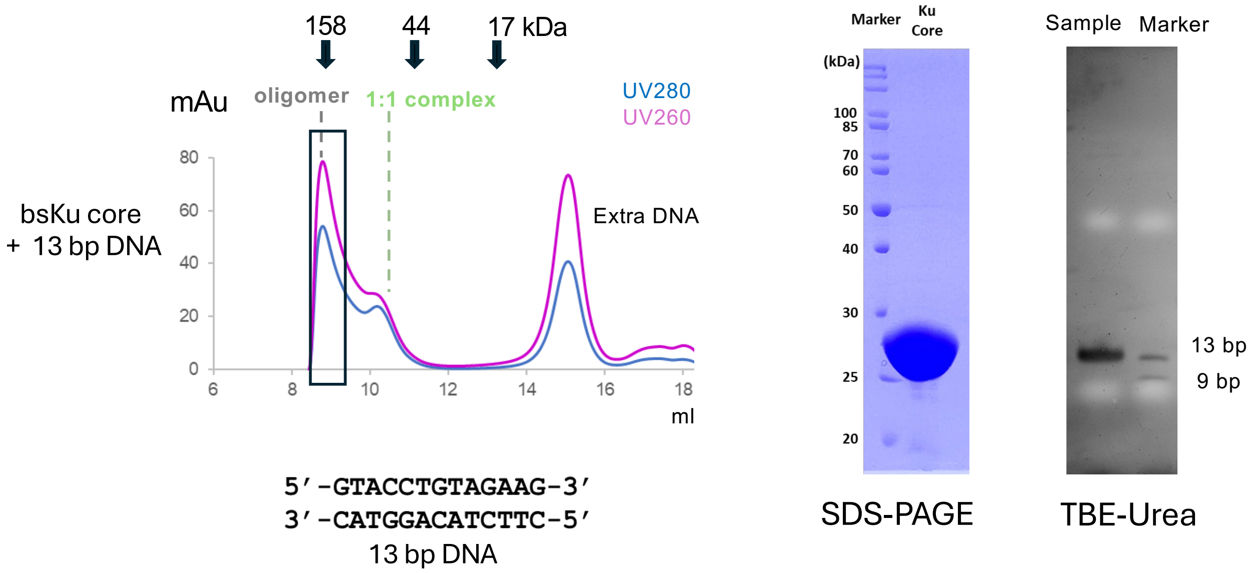

Supplementary Figure 1. Purification of the bsKu core protein-13 bp DNA synaptic complex. Size-exclusion chromatography (left) of the bsKu core protein-13 bp DNA complex. Elution positions of molecular weight standards are marked with arrows at the top of the chromatograph (158 kDa = γ-globulin, 44 kDa =ovalbumin, and 17 kDa=myoglobin). The violet and blue lines represent UV absorbance at 260 nm and 280 nm, respectively. The boxed region indicates the complex sample used for cryo-EM analysis. The protein and DNA quality of this sample were confirmed by SDS-PGAE and denaturing polyacrylamide TBE-urea gel analysis, as shown in the middle and right panels.

Supplementary Figure 2

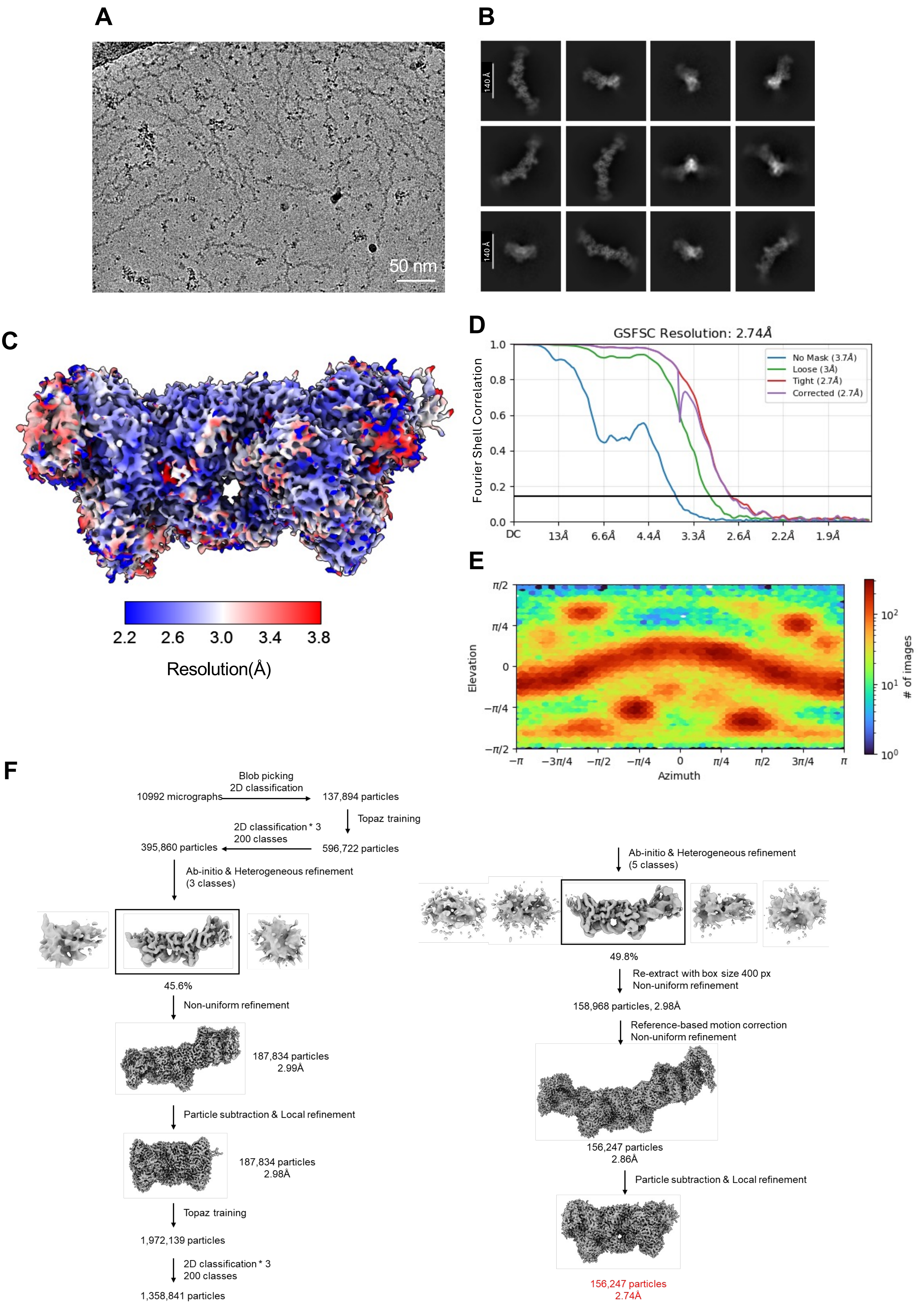

Supplementary Figure 2. Data processing and structure refinement of the bsKu-DNA synaptic complex. (A) A representative cryo-EM micrograph from a total of 10,992 collected. (B) 2D classification images. Scale bar corresponding to 140 Å (C) Composite map colored according to the resolution scale shown at the bottom. (D) FSC analysis of the data quality and map resolution. (E) Angular distribution of particles used in the final map calculation. (F) Workflow of image processing.

Supplementary Figure 3

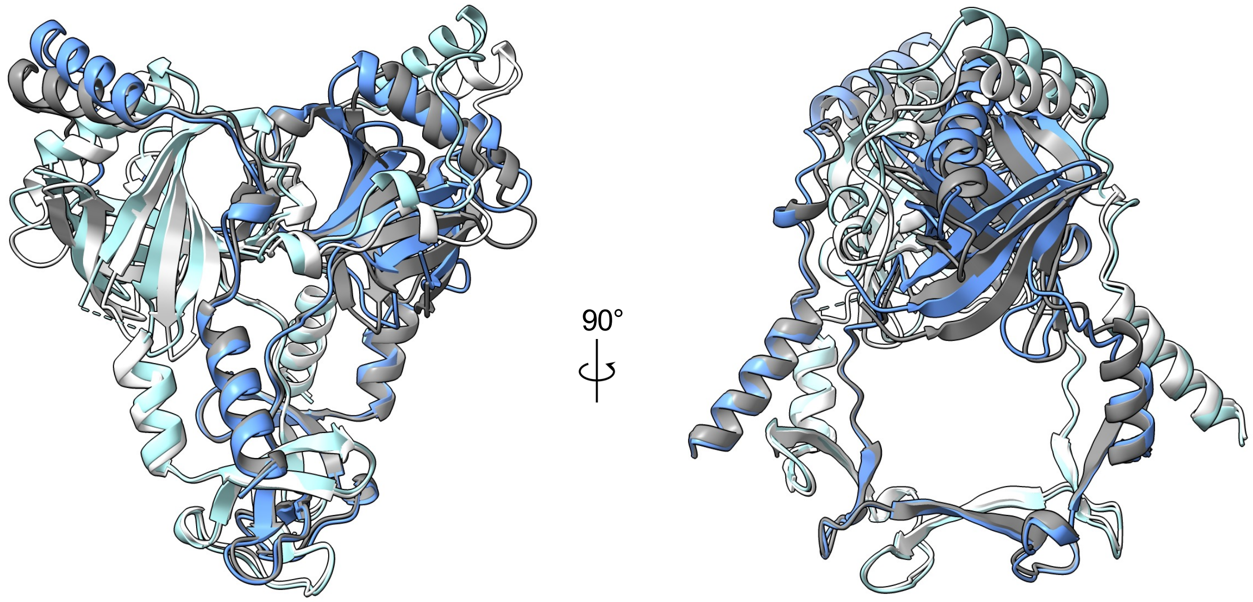

Supplementary Figure 3. Superposition of the cryo-EM structure of the bsKu core domain (residues Met18-Asn244) with the AlphaFold3-predicted model. Each protomer of cryo-EM bsKu homodimer is colored blue and pale blue, respectively, while the corresponding AlphaFold3 model is shown in gray and white. The root-mean-square deviation (RMSD) between the aligned Cα atoms is 2.28 Å.

Supplementary Figure 4

A

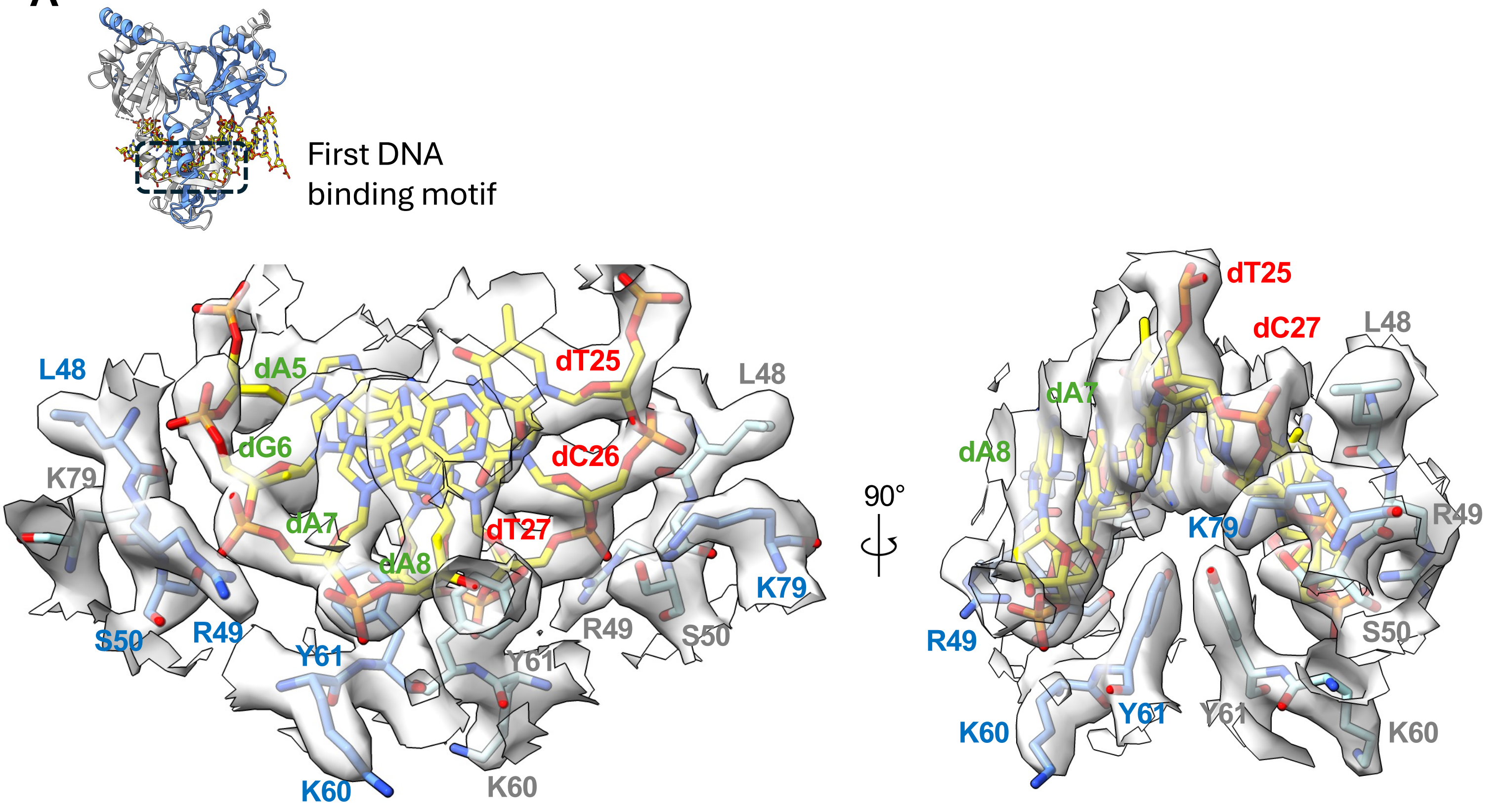

B

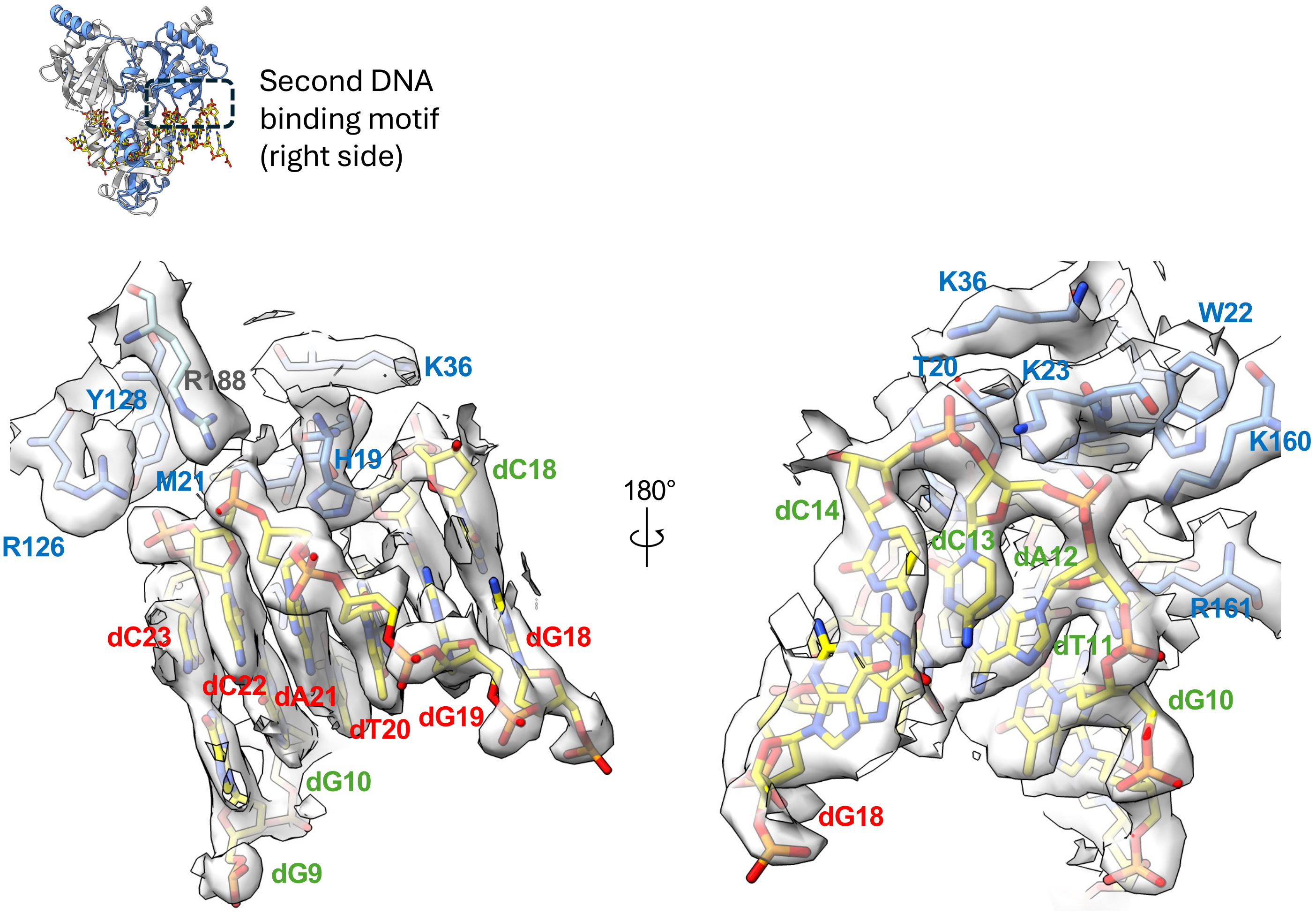

Supplementary Figure 4. Close-up views of the first and second DNA-binding motifs of bsKu. (A) Cryo-EM map of the first DNA-binding motif, with interacting residues highlighted using the same color scheme as Figure 2A. DNA base numbers on the two strands are labeled in green and red, respectively. (B) Cryo-EM map of the second DNA-binding motif, with the interacting residues shown.

Supplementary Figure 5

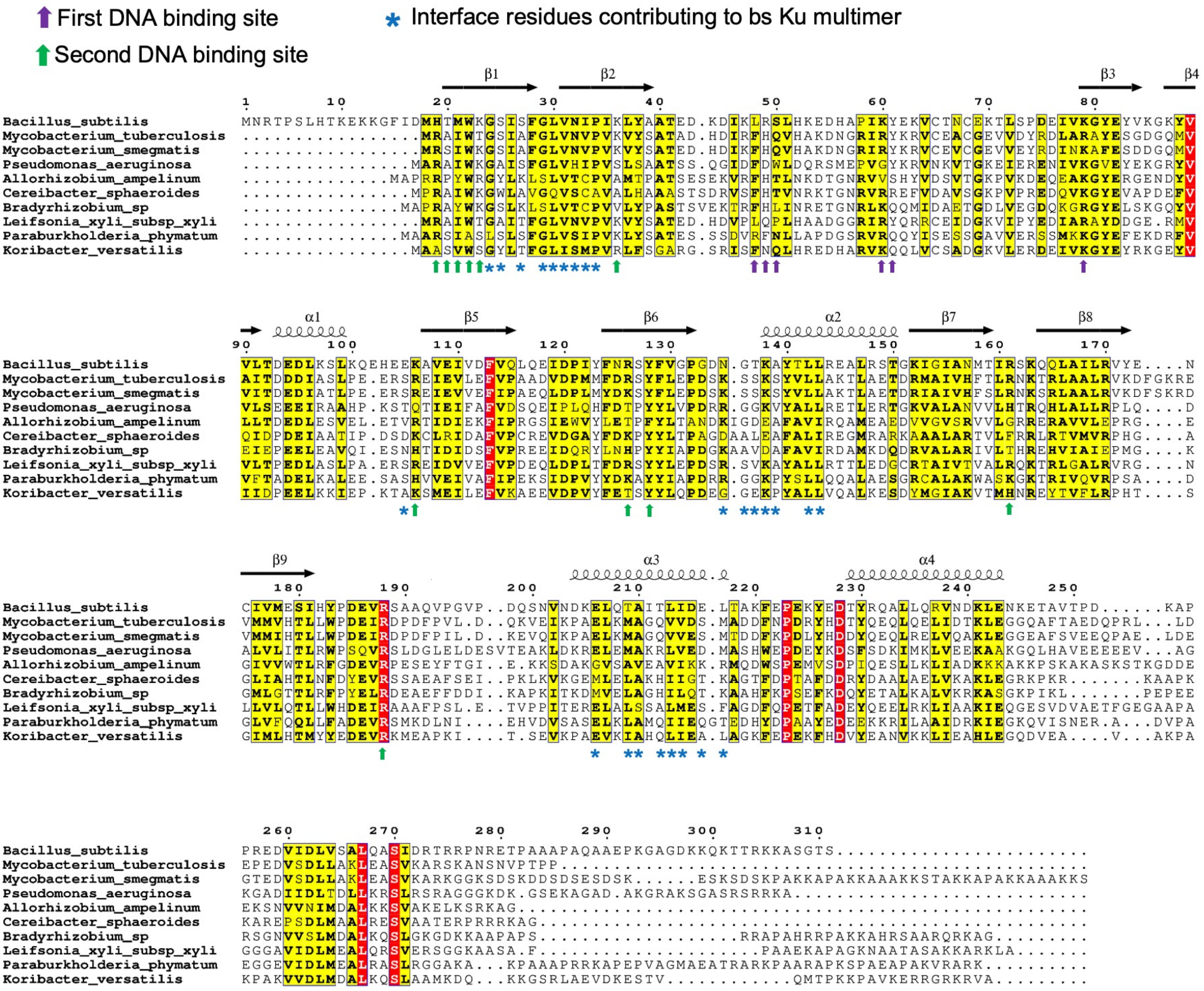

Supplementary Figure 5. Sequence alignment of Ku proteins from various bacterial species. The following species were included in the alignment: *Bacillus subtilis* Ku (UniProt ID: O34859), *Mycobacterium tuberculosis* Ku (UniProt ID: P9WKD9), Mycobacterium smegmatis Ku (UniProt ID: A0R3S7), *Pseudomonas aeruginosa* Ku (UniProt ID: Q9I1W5), *Allorhizobium ampelinum* Ku (UniProt ID: B9K4A6), *Cereibacter sphaeroides* Ku (UniProt ID: B9KKS1), *Bradyrhizobium sp.* Ku (UniProt ID: A4Z012), *Leifsonia xyli subsp. xyli* Ku (UniProt ID: Q6AE23), *Paraburkholderia phymatum* Ku (UniProt ID: B2JMF6), *Koribacter versatilis* Ku (UniProt ID: Q1INE2). Sequence alignment was performed using the Clustal Omega program (1) and visualized with ESPript (2). The secondary structure of bsKu is displayed above the sequence. The first and second DNA-binding sites are indicated by violet and green arrows, respectively, while residues at the bsKu multimer interface are marked with green stars.

Supplementary Figure 6

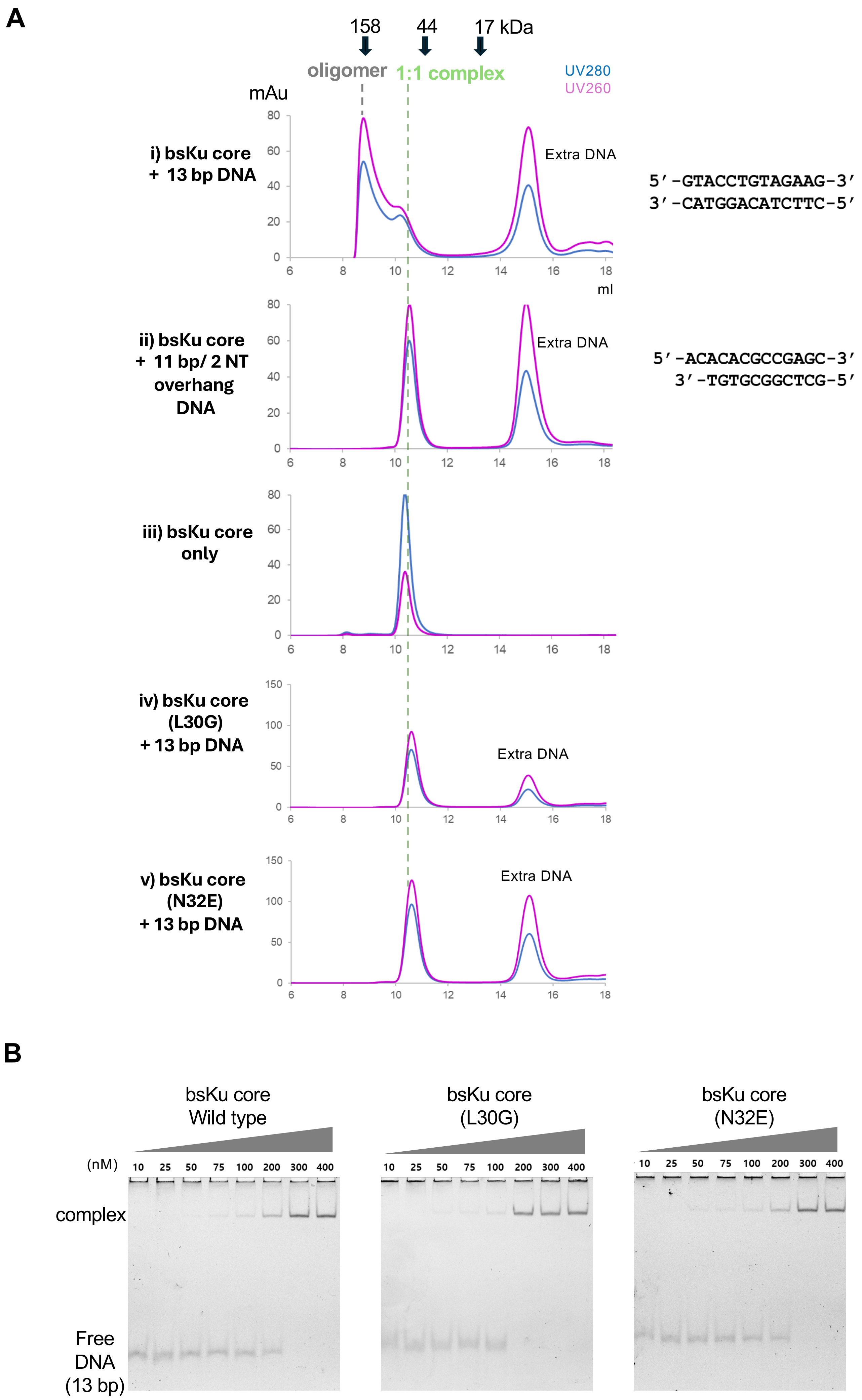

Supplementary Figure 6. DNA-binding analysis of bsKu core and mutants. (A) Size-exclusion chromatography of bsKu wild type and mutants with various DNA substrates. Elution positions of molecular weight standards are marked with arrows at the top of the chromatograph (158 kDa = γ-globulin, 44 kDa =ovalbumin, and 17 kDa=myoglobin). The violet and blue lines represent UV absorbance at 260 nm and 280 nm, respectively. The dashed green line marks the position corresponding to a 1:1 stoichiometry of the bsKu-DNA complex, while the dashed gray line indicates the position of oligomeric bsKu-DNA complexes. i) bsKu core protein with 13-bp DNA ii) bsKu core with an 11 bp DNA containing extra 2-nucleotide overhangs forms a 1:1 complex but does not result in oligomerization. The corresponding DNA sequences are shown to the right. iii) bsKu alone. iv) bsKu L30G mutant with 13-bp DNA. (no oligomerization detected) v) bsKu N32E mutant with 13 bp DNA (No oligomerization detected). (B) EMSA analysis of bsKu core binding to 13-bp DNA, comparing the wild type with two oligomer-interface mutants (L30G and N32E). Protein concentrations are shown above each lane.

Supplementary Figure 7

```
Bacillus_Subtilis_Ku  .....................................................................................
Homo_Sapiens_Ku70     MSGWESYYKTEGDEEAEEEQEENLEASGDYKYSGRDS.LIFLVDASKAMFESQSEDELTPFDMSIQCIQSVYISKIISSDRDLLA
Homo_Sapiens_Ku80     .............................MVRSGNKAAVVLCMDVGFTMSN.SIPGIESPFEQAKKVITMFVQRQVFAENKDEIA

Bacillus_Subtilis_Ku  .....................................................................................
Homo_Sapiens_Ku70     VVFYGTEKDKNSVNFK....NIYVLQELDNPGAKRILELDQFKGQQ..GQKRFQDMMGHGSDYSLSEV.....LWVCANLFSDVQ
Homo_Sapiens_Ku80     LVLFGTDGTDNPLSGGDQYQNITVHRHLMLPDFDLLEDIESK.IQPGSQQADFLDALIVSMDVIQHETIGKKFEKRHIEIFTDLS

Bacillus_Subtilis_Ku  .....................................................................................
Homo_Sapiens_Ku70     FKMSHKRI.................MLFT.NEDNPHGNDSAKASRARTKAGDLRDTGIFLDLMHLKKPGGFDISLFYRDIISIAE
Homo_Sapiens_Ku80     SRFSKSQLDIIIHSLKKCDISLQFFLPFSLGKEDGSGDRGDGPFRLGGHGPSFPLKGI.....TEQQKEGLEI..VKMVMISLEG

                                1       10        20        30         40               50        60
Bacillus_Subtilis_Ku  ..........MNRTPSLHTKEKKGFIDMHTMWKGSISFGL.VNIPIKLYAATEDKDI.......KLRSLHKEDHAPIKYEKVCTN
Homo_Sapiens_Ku70     DEDLRVHFEESSKLEDLLRKVRAKETRKRALSRLKLKLNKDIVISVGIYNLVQKALK......PPPIKLYRETNEPVKTKTRTFN
Homo_Sapiens_Ku80     EDGLDEIYSFSESLRKLCV..FKKIERHSIHWPCRLTIGSNLSIRIAAYKSILQERVKKTWTVVDAKTLKKEDIQK..ETVYCLN

                         70        80        90       100       110       120        130
Bacillus_Subtilis_Ku  C..EKTLSPDEIVKGYEYVKGKYVVLTDEDLKSLKQEHEEKAVEIVDFVQLQEID.PIYFNRSYFVGPGDN..........GTKA
Homo_Sapiens_Ku70     TSTGGLLLPSDTKRSQIYGSRQIILEKEETEELKRFD..DPGLMLMGFKPLVLLKKHHYLRPSLFVYPEESLVIGS......STL
Homo_Sapiens_Ku80     DDDETEVLKEDIIQGFRYGSDIV.PFSKVDEEQMKYKSEGKCFSVLGFCKSSQVQ......RRFFMGNQVLKVFAARDDEAAAVA

                    140       150       160                     170       180       190        200
Bacillus_Subtilis_Ku  YTLLREALRSTGKIGIANMTIRSKQQL......A........ILRVYENCIVMESIHYPDEVRSAAQVPGVP.DQSNVNDKELQT
Homo_Sapiens_Ku70     FSALLIKCLEKEVAALCRYTPRRNIPPYFVALVPQEEELDDQKIQVTPPGFQLVFLPFADDKRKMPFTE.....KIMATPEQVGK
Homo_Sapiens_Ku80     LSSLIHALDDLDMVAIVRYAYDKRANPQVGVAFP........HIKHNYECLVYVQLPFMEDLRQYMFSSLKNSKKYAPTEAQLNA

                    210                   220       230       240       250         260       270
Bacillus_Subtilis_Ku  AITLIDELT............AKFEPEKYEDTYRQALLQRVNDKLENKETAVTPDK..APPREDVIDLVSALQASIDRTR.RPNR
Homo_Sapiens_Ku70     MKAIVEKLR............FTYRSDSFENPVLQQHFRNLEALA......LDLMEPE.....QAVDLTLPKVEAMNKRLGSLVD
Homo_Sapiens_Ku80     VDALIDSMSLAKKDEKTDTLEDLFPTTKIPNPRFQRLFQCLLHRA......LHPREPLPPIQQHIWNMLNPPAEVTTKSQ.IPLS

                    280                      290       300       310
Bacillus_Subtilis_Ku  ETPAAAPA...............QAAEPKGAGDKKQKTTRKKASGTS......................................
Homo_Sapiens_Ku70     EFKELVYPPDYNPEGKVT....KRKHDNEGSGSKRPKVEYSEEELKTHISKGTLGKFTVPMLKE..ACRAYGLKSGLKKQELL..
Homo_Sapiens_Ku80     KIKTLFPLIEAKKKDQVTAQEIFQDNHEDGPTAKKLKTEQGG....AHFSVSSLAEGSVTSVGSVNPAENFRVLVKQKKASFEEA

Bacillus_Subtilis_Ku  .....................................................................................
Homo_Sapiens_Ku70     .EALTKHFQD...........................................................................
Homo_Sapiens_Ku80     SNQLINHIEQFLDTNETPYFMKSIDCIRAFREEAIKFSEEQRFNNFLKALQEKVEIKQLNHFWEIVVQDGITLITKEEASGSSVT

Bacillus_Subtilis_Ku  ...................................
Homo_Sapiens_Ku70     ...................................
Homo_Sapiens_Ku80     AEEAKKFLAPKDKPSGDTAAVFEEGGDVDDLLDMI
```

Supplementary Figure 7. Sequence alignment of bsKu, human Ku70, and human Ku80. Resiude numbering corresponds to the bsKu protein sequence.

Supplementary Figure 8

A

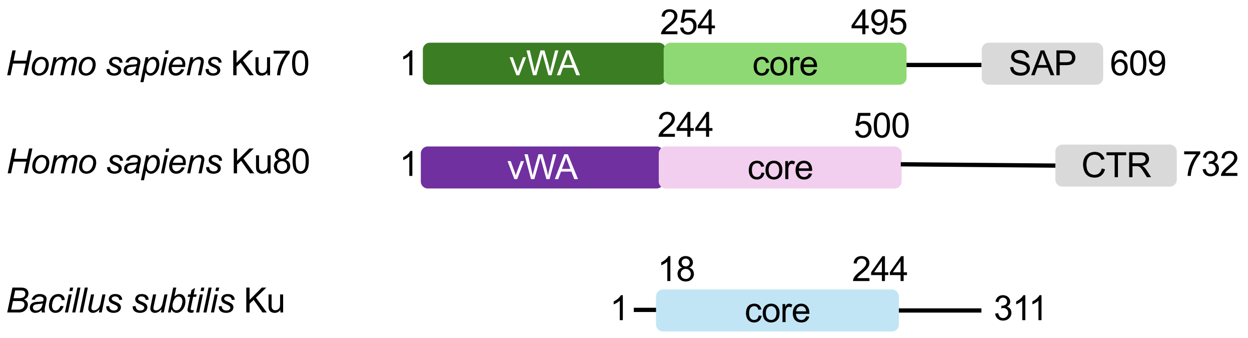

B

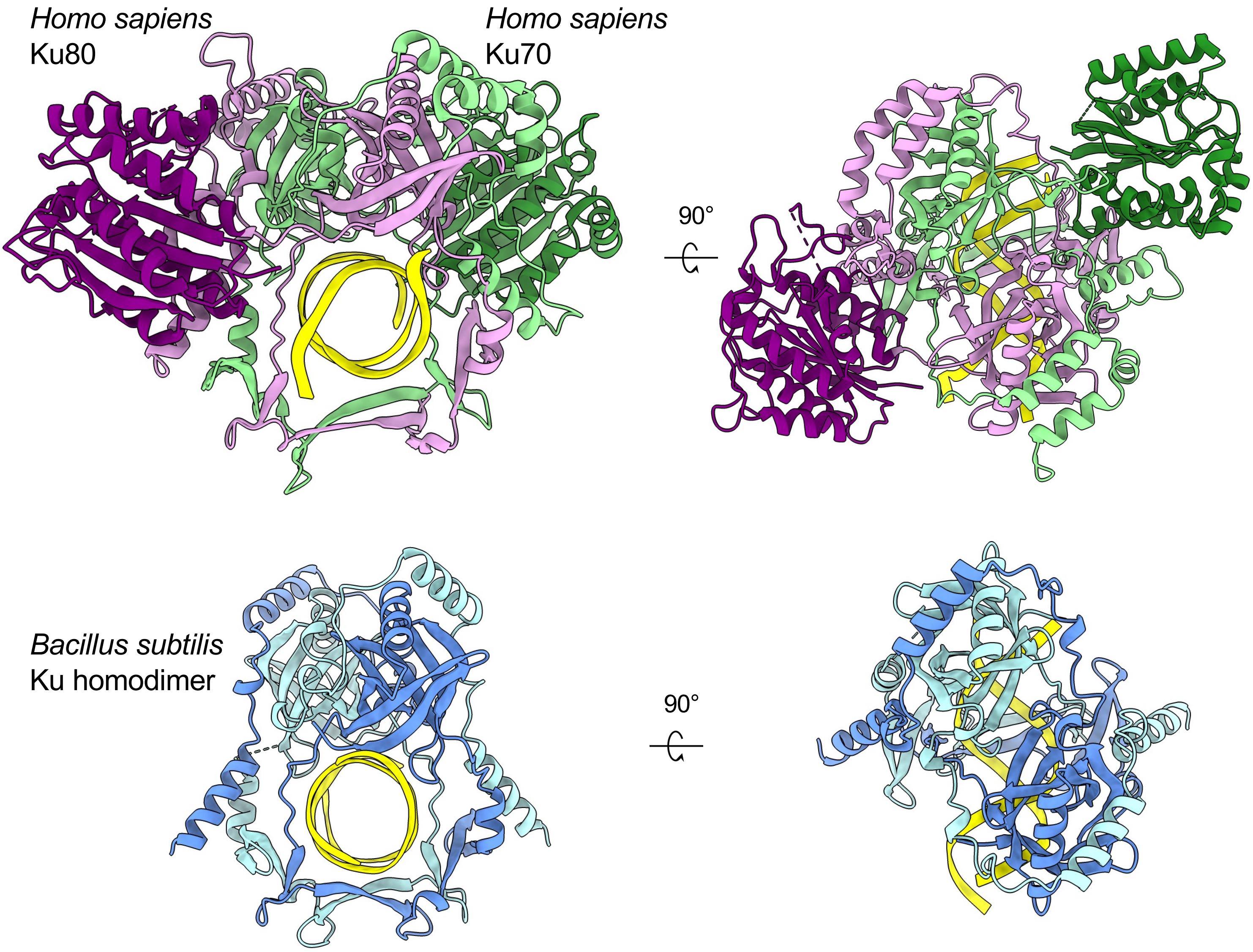

Supplementary Figure 8. Structural comparison between the human Ku70-Ku80 heterodimer and the bsKu homodimer. (A) Domain organization of human Ku70, Ku80, and bsKu. (B) Overall structures of human Ku70-Ku80 heterodimer-DNA complex (PDB ID: 1JEY) and the bsKu-DNA complex (this study, PDB ID: 9VNQ). Both structures are shown in the same orientation. Domain color schemes correspond to those in the domain organization diagram in panel A.

Supplementary Table 1. Cryo-EM data collection, refinement, and validation for bsKu-DNA synaptic complex.

	Bacillus subtilis Ku-DNA synaptic complex (EMD-65215) (PDB 9VNQ)
**Data collection and processing**	
Microscope	Titan Krios G4
Detector	Gatan K3
Magnification	105 k
Voltage (kV)	300
Electron exposure (e–/Å^2)	60
Defocus range (μm)	-0.8 to -2.0
Pixel size (Å)	0.8248
Symmetry imposed	C1
Initial particle images (no.)	1,972,139
Final particle images (no.)	156,247
Map resolution (Å)	2.74
FSC threshold, 0.143	
**Refinement**	
Initial model used	Alphafold3 prediction
Model composition	
Non-hydrogen atoms	8559
Protein residues	905
Nucleotides	62
B factors (Å^2)	
Protein	46.79
Nucleotides	62.63
R.m.s. deviations	
Bond lengths (Å)	0.002
Bond angles (°)	0.497
Validation	
MolProbity score	1.28
Clashscore	5.19
Poor rotamers (%)	0.98
Ramachandran plot	
Favored (%)	98.66
Allowed (%)	1.34
Disallowed (%)	0

Supplementary Table 2. DNA oligonucleotides used for bsKu binding analysis.

Name	Sequence	Length (nt)	DNA type
BAKU_1	GAAGTACCTGTAGAAGC	17	17 bp dsDNA
BAKU_2	GCTTCTACAGGTACTTC	17	
BAKU_3	GAGTACCTGTAGAAG	15	15 bp dsDNA
BAKU_4	CTTCTACAGGTACTC	15	
BAKU_5	GTACCTGTAGAAG	13	13 bp dsDNA
BAKU_6	CTTCTACAGGTAC	13	
BAKU_7	CCTTACGTGCCC	12	12 bp dsDNA
BAKU_8	GGGCACGTAAGG	12	
BAKU_9	CCGACGTGCC	10	10 bp dsDNA
BAKU_10	GGCACGTCGG	10	
BAKU_11	CCTTCGTGCCC	11	11 bp dsDNA
BAKU_12	GGGCACGAAGG	11	
BAKU_13	GGC ACG AGG	9	9 bp dsDNA
BAKU_14	CCT CGT GCC	9	
BAKU_15	CGC CGA GC	8	8 bp dsDNA
BAKU_16	GCT CGG CG	8	
BAKU_17	ACCTTCGTGCCC	12	11 bp dsDNA + 1 nt 5' overhang
BAKU_18	TGGGCACGAAGG	12	
BAKU_19	A CCGACGTGCC	11	10 bp dsDNA + 1 nt 5' overhang
BAKU_20	T GGCACGTCGG	11	
BAKU_21	A GGC ACG AGG	10	9 bp dsDNA + 1 nt 5' overhang
BAKU_22	T CCT CGT GCC	10	
BAKU_23	A CGC CGA GC	9	8 bp dsDNA + 1 nt 5' overhang
BAKU_24	T GCT CGG CG	9	
BAKU_25	ACACACGCCGAGC	13	8 bp dsDNA + 5 nt 5' overhang w/ BAKU_16
BAKU_26	GCTCGGCGTGT	11	8 bp dsDNA + 2 nt 5' overhang w/ BAKU_25
BAKU_27	GCTCGGCGT	9	8 bp dsDNA + 4 nt 5' overhang w/ BAKU_25
BAKU_28	GCTCGGCGTGACC	13	8 bp dsDNA + 5 nt 3' overhang w/ BAKU_15 / 4 nt 3' overhang w/ BAKU_23
BAKU_29	TCACGCCGAGC	11	8 bp dsDNA + 2 nt 3' overhang w/ BAKU_28
BAKU_30	GCCGAG	6	6 bp dsDNA
BAKU_31	CTCGGC	6	
BAKU_32	CCCCC	5	5 bp dsDNA
BAKU_33	GGGGG	5	

Supplementary References

1. Sievers,F. and Higgins,D.G. (2018) Clustal Omega for making accurate alignments of many protein sequences. *Protein Science*, 27, 135–145.

2. Robert,X. and Gouet,P. (2014) Deciphering key features in protein structures with the new ENDscript server. *Nucleic Acids Research, 42*, W320–W324.
